# A Cadaveric Study of the Anatomical Characteristics of the Superficial Circumflex Iliac Artery Perforator

**DOI:** 10.1055/s-0045-1802643

**Published:** 2025-02-11

**Authors:** Tanvi Rao, Vijay Jaganathan, Jonathan Victor, Pappu Paramartha Lingam

**Affiliations:** 1 Department of Plastic Surgery, Kasturba Medical College, Manipal, Manipal Academy of Higher Education, Manipal, Karnataka, India; 2 Department of Plastic Surgery, Pondicherry Institute of Medical Sciences, Puducherry, India; 3 Department of Plastic Surgery, Christian Medical College, Vellore, Tamil Nadu, India

**Keywords:** SCIP flap, cadaveric study, perforators, superficial circumflex iliac artery, anatomic variations

## Abstract

**Background** The superficial circumflex iliac artery perforator flap (SCIP flap) is an evolution of the conventional free groin flap. Even though the anatomical basis for SCIP flap is well established in general, the same is not described in a South Indian population.

**Objectives** The objectives of our study were to determine the anatomical variations of the superficial circumflex iliac artery perforators (SCIA perforators) and describe them in relation to the nearby bony landmarks like anterior superior iliac spine (ASIS) and pubic tubercle (PT).

**Materials and Methods** This observational study was done between October 2020 and December 2021. Cadaveric dissection was done and the required anatomic data were collected.

**Results** The mean diameter of SCIA was 0.99 mm and the mean pedicle length was 2.97 cm. The average diameters of the medial and lateral perforators were 0.63 and 0.55 mm, respectively. The pedicle lengths of the medial and lateral perforators were 3.03 and 4.31 cm, respectively. The medial perforator was 5.63 cm lateral and 1.66 cm superior to the PT. It was 5.37 cm medial and 5.99 cm inferior to the ASIS. The lateral perforator was 7.97 cm lateral and 2.73 cm superior to the PT. It was 2.86 cm medial and 4.11 cm inferior to the ASIS.

**Conclusion** Finding the exact location of the medial and the lateral perforators with respect to permanent bony landmarks and having an idea about their average pedicle length are useful for preoperative planning of the SCIP flap.

## Introduction

Reconstruction of large composite soft tissue defects is currently done by free tissue transfers. The superficial circumflex iliac artery perforator flap (SCIP flap) is an evolution of the conventional free groin flap. 1 The groin flap is based on the superficial circumflex iliac artery (SCIA). It was first described by McGregor and Jackson in 1972. The groin flap became popular due to the concealment of the donor site scar and availability of a large cutaneous flap. But recently, to overcome the shortcomings of the groin flap (such as arterial anatomical variation, a short pedicle, and the bulkiness of the flap), new flaps are being studied and their use is being researched. The development of perforator flaps (PFs) has enabled the use of thinner flaps and progress in imaging provides a reliable identification of vessels that supply the flaps. The PFs based on the SCIA have been described for local or distant coverage of wounds.

With the recent development of PFs such as SCIP flaps, the major pedicle vessels of fasciocutaneous flaps could be left in place at the donor site. Instead, the flap can be harvested based on the branches of the major pedicle, thus minimizing donor site morbidity and preserving reliable blood supply to the flap. 2

The use of SCIP flap has gradually increased in the reconstruction of small to medium sized defects in the extremities. Mostly, the SCIP flap is perfused only by the superficial branch of SCIA. But the deep branch of SCIA is included in the flap when a large flap is required for coverage or when one of the following anatomical structures perfused by the deep branch is elevated with the skin paddle: the sartorius muscle, the iliac bone, and the lateral femoral cutaneous nerve. 3

The SCIA arises from the femoral artery. It is the smallest superficial branch of the femoral artery and originates very close to the superficial inferior epigastric artery (SIEA). The average length of SCIA is 2 cm (range: 1.5–3 cm) and the average diameter is 1.5 mm (range: 0.8–2 mm). 4

The SCIA arises about 3 cm below the inguinal ligament from the femoral artery and runs laterally. It may have a superficial and/or deep branch. The superficial branch, when present, runs superolaterally over the sartorius fascia, giving few perforators, while the deep branch runs beneath the sartorius fascia, penetrating at its lateral border to give perforators in the anterolateral groin. It penetrates the deep fascia at the lateral border of the sartorius muscle and then enters the suprafascial layer. This cadaveric study aimed to determine the diameter, location, and reliability of perforators arising from the SCIA and to describe the existence and the anatomical location of its branches. We aim to explore the anatomical variations, if any, of the SCIA perforators in a South Indian population to assess if these findings could improve the accuracy of surface markings for the SCIP flap in individuals with similar anthropometric characteristics.

## Materials and Methods

This was an observational study which was conducted after obtaining institutional committee clearance [IEC Ref No: RC/2020/84]. Data were collected between October 2020 and December 2021. Cadavers satisfying the inclusion criteria were used for the study. Twenty cadavers dissected bilaterally were analyzed. Twenty cadavers with no scars in the groin region were included in the study.

The following parameters were studied:

- Number of perforators identified from SCIA.
- Origin of SCIA.
- Diameter of perforators.
- Medial and lateral perforator details.
- Distance of each perforator from anterior superior iliac spine (ASIS).
- Distance of each perforator from pubic tubercle (PT).
- Distance of each perforator from femoral artery.
- Distance between ASIS and PT, inter-ASIS and inter-PT distance.
- Relationship of perforator to mid-clavicular line (MCL).
- Course of lateral perforator—direct cutaneous or intramuscular.

### Procedure Details

**Fig. 1:**
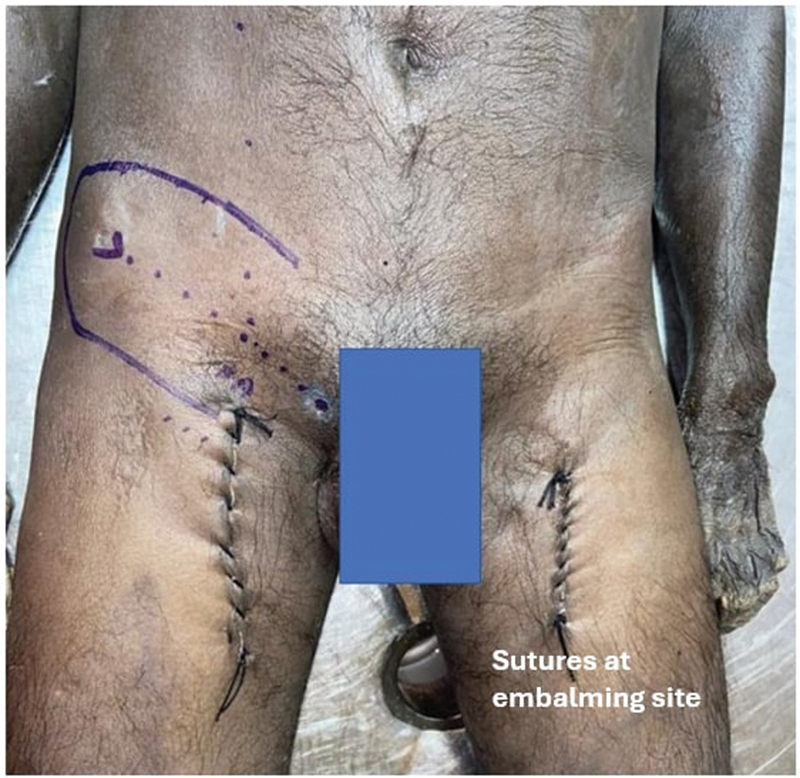
Markings of the bony landmarks. A, surface marking of SCIA origin; ASIS, anterosuperior iliac spine; PT, pubic tubercle.

**Fig. 2:**
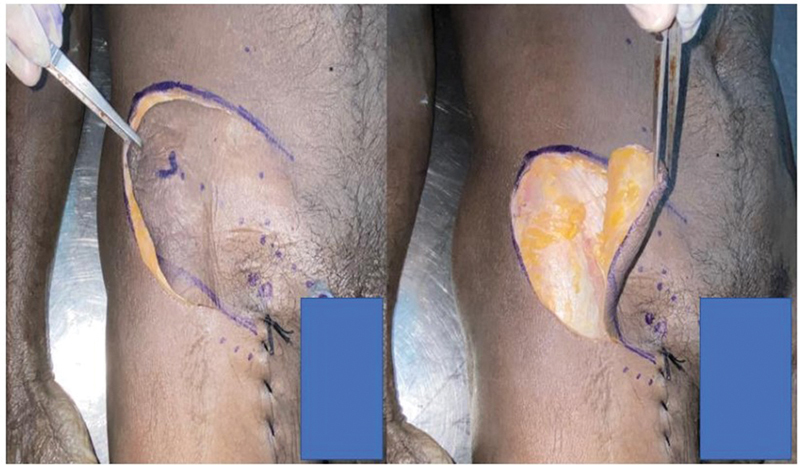
Raising the SCIP flap from lateral to medial direction. SCIP, superficial circumflex iliac artery perforator.

**Fig. 3:**
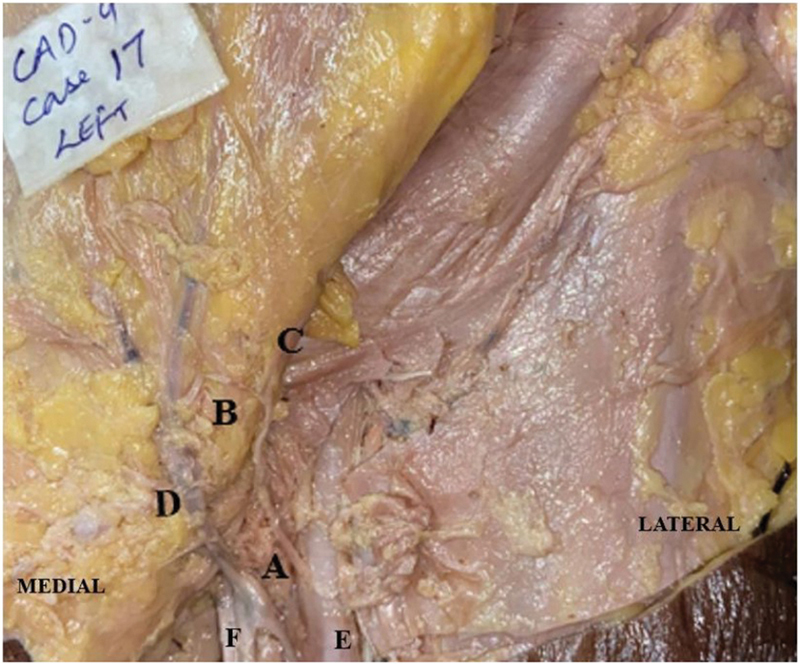
Image showing the (A) SCIA originating from the superficial femoral artery, (B) the medial perforator of the SCIA, (C) the lateral perforator of the SCIA, (D) the superficial circumflex iliac vein from the greater saphenous vein, (E) superficial femoral artery, and (F) the femoral vein. SCIA, superficial circumflex iliac artery.

**Fig. 4:**
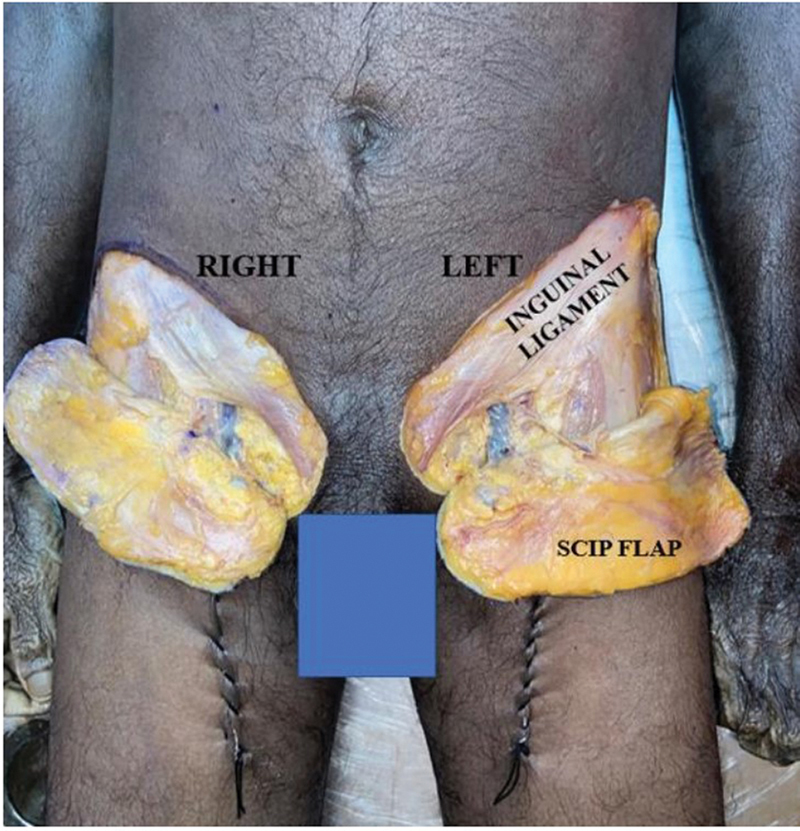
Bilateral SCIP flaps raised in the cadaver. SCIP, superficial circumflex iliac artery perforator.

Twenty cadavers were dissected and the SCIP flap was raised bilaterally. The ASIS, the PT, and the MCL were used as skin surface landmarks ( Fig. 1 ). The SCIA typically originates 2.5 cm below the midpoint of the inguinal ligament and then runs parallel to it. Dissection was done from lateral to medial direction and proceeded from the inferolateral part of the groin until the perforators were identified ( Fig. 2 ). Superomedial incision was committed, and careful dissection was done to identify perforators from other vessels like SIEA or superficial external pudendal artery and to locate the veins ( Figs. 3 and 4 ). The anatomic dimensions of the perforators were noted and the parameters mentioned above were recorded using a digital Vernier calliper with a precision of 0.01 mm.

The data were entered in Microsoft Excel and statistically analyzed using the SPSS software version 20.0. Descriptive statistics was used to analyze the result. For categorical variables, frequencies and percentages were applied. For the continuous variables, mean and standard deviations were calculated. As the study did not involve living participants, waiver of consent was obtained from the ethical committee.

## Results

**Fig. 5:**
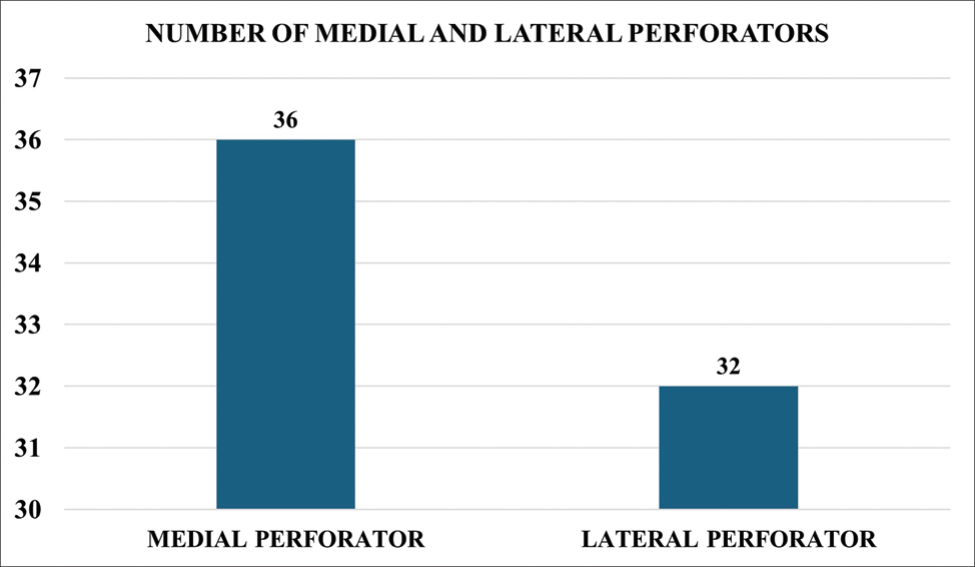
Number of medial and lateral perforators.

Out of the 40 cadavers dissected, one perforator was noted in 12/40 cases and both perforators were present in 28/40 cases. Among these, medial perforators were present in 36/40 cases and lateral perforators in 32/40 cases ( Fig. 5 ).

**Fig. 6:**
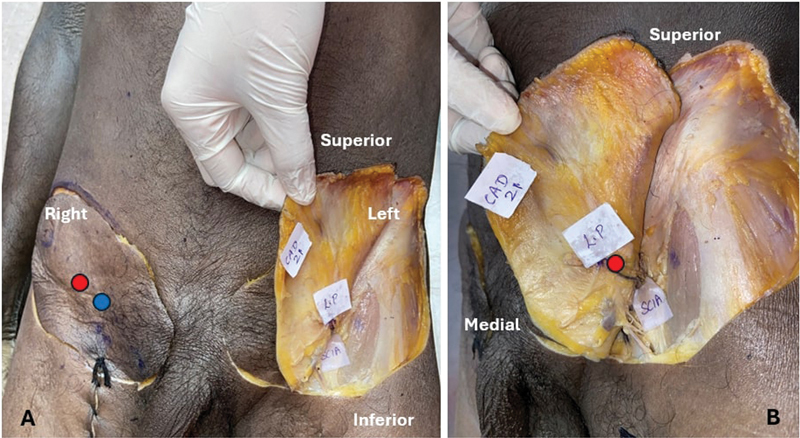
( **A** ) Example of the anatomic variations: presence of both the perforators (marked by the red and blue dots) on the right side; presence of only the lateral perforator on the left side. ( **B** ) Dissected cadaver showing the SCIA and the lateral perforator and absent medial perforator. SCIA, superficial circumflex iliac artery.

**Fig. 7:**
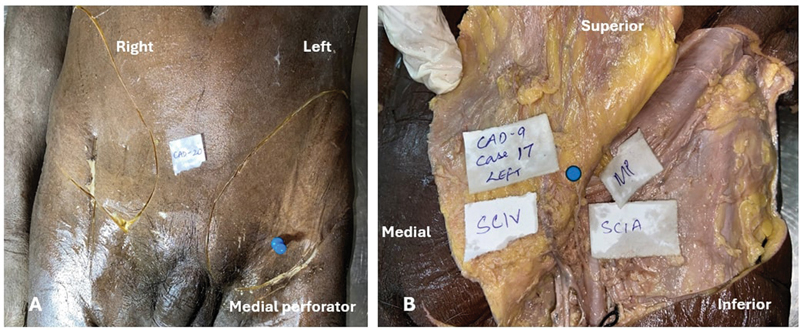
Example of an anatomic variation. ( **A** ) Left side of a cadaveric dissection showing the presence of only the medial perforator (blue pin). ( **B** ) Cadaveric dissection showing only the medial perforator (blue dot), SCIA, and absence of the lateral perforator. SCIA, superficial circumflex iliac artery.

The presence or absence of the perforators varied. The medial and lateral perforators were both present in most cases (28/40). In some, only the medial perforators were noted (8/40), whereas in others only the lateral perforator was present (4/40; Figs. 6 and 7 ).

The origin of the SCIA noted in our study was from either the superficial femoral artery (SFA) or the profunda femoris. The majority of the SCIA originated from the SFA ( *n* = 38, 95%). Only in one cadaver the origin of the SCIA was from the profunda femoris ( *n* = 2, 5%).

The mean diameter of the SCIA was observed to be 0.99 ± 0.51 mm and the mean pedicle length of the SCIA was 2.97 ± 1.46 cm ( Table 1 ).

**Table 1:** Dimensions of SCIA and the perforators

	SCIA	Medial perforator	Lateral perforator
**Size (in mm)**	0.99 ± 0.51	0.63 ± 0.42	0.55 ± 0.36
**Length (in cm)**	2.97 ± 1.46	3.03 ± 1.54	4.31 ± 1.84

Abbreviation: SCIA, superficial circumflex iliac artery.

The mean diameter of the medial perforator was 0.63 mm and of the lateral perforator was 0.55 mm, with a standard deviation of 0.42 and 0.36 mm, respectively. The mean length of the medial perforator is 3.03 cm and of the lateral perforator is 4.31 cm, with a standard deviation of 1.54 and 1.84 cm, respectively ( Table 1 ).

The diameter of the perforators were compared when only one of the perforators was absent or when both the perforators were present. The average diameter of the medial perforator when the lateral perforator was absent was 0.55 mm and its average diameter was 0.64 mm when both the perforators were present. The average diameter of the lateral perforator when the medial perforator was absent was 0.41 mm and its average diameter was 0.57 mm when both the perforators were present.

The distance of the medial and lateral perforators from bony landmarks was noted and analyzed. The mean distance from each bony landmark was recorded. The medial perforator was 5.63 cm lateral and 1.66 cm superior to the PT and 5.37 cm medial and 5.99 cm inferior to the ASIS. The lateral perforator was at a mean distance of 7.97 cm lateral and 1.95 cm superior to the PT, and 2.86 cm medial and 4.11 cm inferior to the ASIS ( Table 2 ).

**Table 2:** Distance of perforators from bony landmarks

Distance (cm), mean ± SD	Medial perforator	Lateral perforator
**Lateral to PT**	5.63 ± 1.56	7.97 ± 1.95
**Superior to PT**	1.66 ± 1.34	2.73 ± 1.46
**Medial to ASIS**	5.37 ± 1.53	2.86 ± 1.69
**Inferior to ASIS**	5.99 ± 2.08	4.11 ± 1.67

Abbreviations: ASIS, anterior superior iliac spine; PT, pubic tubercle; SD, standard deviation.

There are two types of travel of the medial perforator within the flap—direct anchorage after it arises from the SCIA and the other travels for some distance before entering the flap. In our study, we noted that 55.5% ( *n* = 20/36) of the medial perforators showed axial pattern travel and 44.46% ( *n* = 16/36) showed direct cutaneous pattern of travel.

Among the 40 dissections done, lateral perforator was found in 32 cases. The lateral perforator was found to be giving muscular branches to the sartorius in *n* = 27 (84%) cases. No muscular branches were noted in *n* = 5 (16%) cases.

**Fig. 8:**
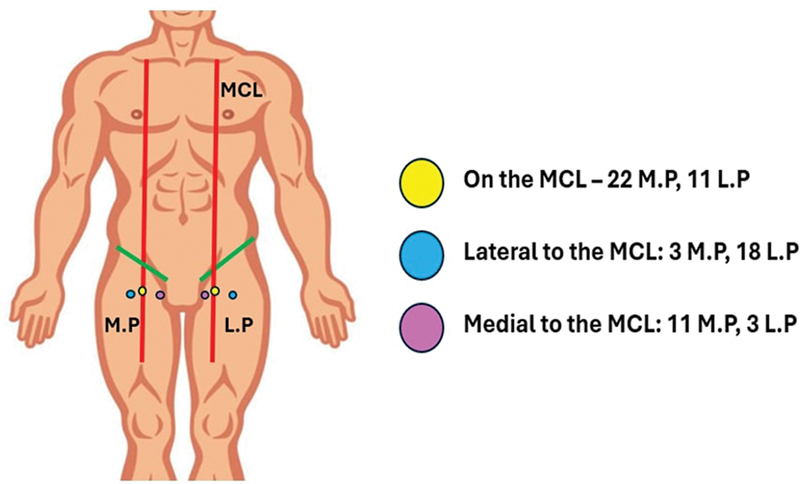
Number of medial perforators (M.P) and lateral perforators (L.P) located medial to, lateral to, or on the mid-clavicular line (MCL).

The relationship of the medial and lateral perforators in relation to the MCL was observed and noted. It was seen that 22 out of the 36 medial perforators (61.11%) were present on the MCL, 11 (30.56%) were medial only, and 3 (8.33%) were lateral to the MCL ( Fig. 8 ).

In total, 11 out of the 32 (34.38%) lateral perforators were on the MCL, 3/32 (9.38%) were medial, and 18/32 (56.25%) were lateral to the MCL.

**Fig. 9:**
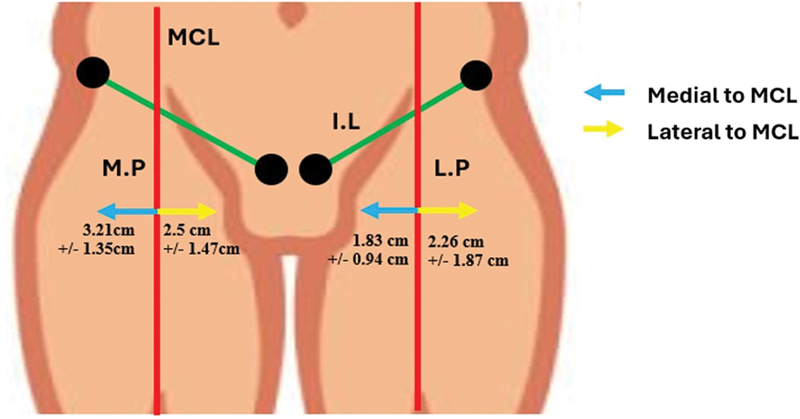
Distance of the medial perforator (M.P) and lateral perforator (L.P) from the mid-clavicular line (MCL).

Excluding the perforators that were lying on the MCL, the mean distance of the medial perforator was 3.21 ± 1.35 cm medial to MCL and that of the lateral perforators was 2.26 ± 1.87 cm lateral to it ( Fig. 9 ).

The mean distance between the PT and the ASIS is 13.41 cm (standard deviation: 1.24 cm). The mean inter-ASIS distance and inter-PT distance are 25.64 and 8.11 cm, respectively (standard deviation: 2.56 and 1.38 cm).

## Discussion

Koshima et al observed that a large groin flap can be raised based on a single, dominant perforator from the deep branch of SCIA. 5 This study aimed to observe the anatomical landmarks of the medial and lateral perforators of the SCIA system in the groin area in the Indian population. Given the lack of data on the SCIA perforators in the Indian population, our study sought to: (1) determine the existence and anatomical variations of perforators arising from the SCIA and its accompanying veins, and (2) describe these perforators in relation to the ASIS and PT.

Notably, data on the location and branching patterns of SCIA perforators in the Indian population have been previously unavailable. Therefore, this study helps fill a knowledge gap in the existing literature. Among the 40 dissections conducted in our study, either medial or lateral perforators were consistently present. The medial perforator was present in 36 out of 40 cases, while the lateral perforator was present in 32 out of 40. In 28 dissections, both medial and lateral perforators were identified, whereas only the medial perforator was present in 8 cases and only the lateral perforator in 4 cases.

Sinna et al 4 noted in their study that a dominant perforator was always present through the sartorius muscle. In contrast in our study, the lateral perforator did not pass through the sartorius muscle in any of the cases. But we noted that the lateral perforator gave muscular branches in 84% of the cases (27/32). We noted that the mean diameter of the SCIA was 0.99 cm ± 0.51 mm. The mean length of the SCIA before it branches into the medial and the lateral perforators was found to be 2.97 ± 1.46 cm. In a study by Kosba et al, 6 the mean diameter of SCIA was noted to be 1.5 ± 0.7 mm and the mean length of the pedicle that could be dissected was 3.2 ± 0.8 cm. In another cadaveric study by Sinna et al, 4 the mean diameter of the SCIA was found to be 1.92 ± 0.6 mm and the mean pedicle length of the SCIA was 4.8 ± 1.3 cm. The mean diameter of the medial and lateral perforators in our study was observed to be 0.63 and 0.54 mm, respectively. In a cadaveric study by Yoshimatsu et al, 3 the diameter of the medial perforator of the SCIA ranged from 0.5 to 1.2 mm with a mean diameter of 0.9 mm. The diameter of the lateral perforator of the SCIA ranged from 0.6 to 2.0 mm with a mean diameter of 1.0 mm. In a cadaveric study done by Gandolfi et al, 7 the mean diameter of the medial perforator was found to be 2.0 ± 0.78 mm and of the lateral perforator was 2.1 ± 0.62 mm. The calibers of the perforators in the above study are larger than the calibers noted in our study. One of the reasons for the difference in calibers is the variation of the body habitus in the population studied. The other reason is the use of embalming via femoral canulation done to preserve the cadavers used in our study. In a study done by Gentileschi et al, 8 they observed that the diameter of the medial perforator ranged from 1 to 2 mm, with a mean of 1.55 ± 0.25 mm. The diameter of the lateral perforator ranged from 0.7 to 1.8 mm, with a mean of 1.33 ± 0.26 mm. The perforators in this study are of larger caliber compared to the perforator diameters in our study. This is because our study is a cadaver-based one, whereas the study by Gentileschi et al is a surgical and radio-anatomical one. 8 In our study, the mean length of the medial and lateral perforator was found to be 3.03 and 4.31 cm, respectively. In a cadaveric study done by Gandolfi et al, 7 the mean length of the medial and lateral perforator was found to be 1.8 ± 0.6 mm and 1.43 ± 0.33 mm, respectively. In our study, the origin of the SCIA was found to be from SFA or from the profunda femoris. In a study by Suh et al, 9 it was found that the SCIA originated from the femoral artery (84.8%). The SCIA may also arise from the SFA (7.4%), the deep femoral artery (6.7%), and the lateral circumflex femoral artery (1.1%). This is in contrast to our findings, in which majority of the SCIA systems originated from the SFA. We noted that the venae comitantes were present with the SCIA in all the dissections. Most of them were collapsed and hence the exact measurements of these veins could not be noted. We noted that there were one to two separate veins along the inferomedial border of the SCIP flap (close to the PT). These veins drained into the greater saphenous vein or into the femoral vein. In some of the cadavers, these veins were sizeable with their diameter ranging between 0.94 and 2.75 mm. A study by Sinna et al showed that the venae comitantes was always smaller (with a mean diameter of 0.73 mm). 4

The findings in our study are in accordance with the results of previous studies and add new information about the position and the course of the SCIA and its perforator. This understanding of the course, diameter, length, and location of the SCIA and its perforators helps in raising the SCIP flap, which is useful for covering a variety of soft tissue defects in the body. Finding the exact location of the medial and the lateral perforators with respect to permanent bony landmarks (PT and ASIS) and having an idea about their average pedicle length are very useful for preoperative planning of the SCIP flap.

The clinical applications of the study include enhanced preoperative planning, more precise flap designing, and reduced operative time. Identifying exact perforator locations also ensures better flap viability and minimizes surgical complications. This study contributes to the anatomical knowledge base, aiding in the education and training of surgeons in flap surgery techniques.

### Limitations

- One limitation of our study is that the analysis was limited to absolute measurements of the SCIA perforators in relation to fixed bony landmarks, such as the ASIS and PT. While this approach provided reliable data, the clinical use of the findings may be increased by expressing perforator locations as ratios or percentages relative to imaginary anatomical lines, such as the MCL or arcs drawn from standardized reference points. The study did not incorporate advanced imaging techniques, such as computed tomography angiography, which could provide a more detailed understanding of the three-dimensional anatomy and relationships of perforators to imaginary anatomical lines or arcs. Such methodologies could account for variations in body habitus and improve preoperative localization in diverse populations. 10 Future studies incorporating imaging modalities, such as computed tomography angiography, may enable the identification of consistent geometric relationships between perforators and imaginary lines, which would aid surgical planning. 11
- The study was conducted on 20 cadavers, resulting in a sample size of 40 dissections. While this is sufficient for preliminary observations, the small sample size limits the generalizability of the findings to the broader South Indian population. Also, the cadavers used in this study may not uniformly represent the entire South Indian population. Variations in factors such as age, sex, and body habitus may affect vascular anatomy but were not analyzed due to the limited demographic information available.
- The study was performed on embalmed cadavers, where vessel diameters and pedicle lengths may differ from in vivo conditions due to the effects of embalming and the absence of blood flow. Also, embalmed veins often collapse or shrink due to absence of blood flow. As a result, the veins were not consistently visible or measurable in all our cases. Therefore, the diameter of the veins could not be reliably assessed in this study. We recommend that future studies utilize fresh cadavers preserved using soft embalming techniques to maintain vascular integrity, such as Thiel embalming. These methods are known to better preserve soft tissue and vascular structures, providing more accurate and reliable anatomical assessments.

### Future Directions

To address these limitations, we recommend larger, multicentric studies involving live imaging techniques and a more diverse sample population. Such studies could explore geometric relationships between perforators and imaginary anatomical lines, improving the clinical utility and applicability of findings in flap design.

## Conclusion

In conclusion, understanding the location of medial and lateral perforators relative to permanent bony landmarks (PT and ASIS), along with their average pedicle length, enhances precision in preoperative planning for raising the SCIP flap. These anatomical details support the effective utilization of the SCIP flap for reconstructing a variety of soft tissue defects.

## References

[1] Strobbe S Van Landuyt K Delaere P Vander Poorten V Vanclooster C Superficial circumflex iliac artery perforator flap for reconstruction of oral defects after tumor resection B-ENT 2015 11 02 157 161 26563018

[2] Koshima I Mardini S Wei F C Groin flap and superficial circumflex iliac artery perforator flap Edinburgh Saunders 2009 359 374

[3] Yoshimatsu H Yamamoto T Hayashi A Use of the transverse branch of the superficial circumflex iliac artery as a landmark facilitating identification and dissection of the deep branch of the superficial circumflex iliac artery for free flap pedicle: anatomical study and clinical applications Microsurgery 2019 39 08 721 729 31591765 10.1002/micr.30518

[4] Sinna R Hajji H Qassemyar Q Perignon D Benhaim T Havet E Anatomical background of the perforator flap based on the deep branch of the superficial circumflex iliac artery (SCIP Flap): a cadaveric study Eplasty 2010 10 e11 20090859 PMC2808053

[5] Koshima I Nanba Y Tsutsui T Superficial circumflex iliac artery perforator flap for reconstruction of limb defects Plast Reconstr Surg 2004 113 01 233 240 14707641 10.1097/01.PRS.0000095948.03605.20

[6] Kosba A Fata M M Borhamy G E Kessler P Habib A S Anatomy of a perforator flap based on the superficial circumflex iliac artery. A cadaveric study Egypt Dent J 2019 65(1-January (Oral Surgery)):89 92

[7] Gandolfi S Postel F Auquit-Auckbur I Vascularization of the superficial circumflex iliac perforator flap (SCIP flap): an anatomical study Surg Radiol Anat 2020 42 04 473 481 31897652 10.1007/s00276-019-02402-9

[8] Gentileschi S Servillo M De Bonis F Radioanatomical study of the pedicle of the superficial circumflex iliac perforator flap J Reconstr Microsurg 2019 35 09 669 676 31315137 10.1055/s-0039-1693144

[9] Suh H SP Jeong H H Choi D H Hong J PJP Study of the medial superficial perforator of the superficial circumflex iliac artery perforator flap using computed tomographic angiography and surgical anatomy in 142 patients Plast Reconstr Surg 2017 139 03 738 748 28234857 10.1097/PRS.0000000000003147

[10] Wong C H Cui F Tan B K Song C In vivo anatomical study and clinical applications of the anterior thigh perforators for perforator flap harvesting Plast Reconstr Surg 2020 145 05 1069 1077

[11] Kim Y H Kim B J Lee J Geometric localization of perforator arteries: a CT angiographic study Ann Plast Surg 2021 86 01 57 62

